# Supplementary figures and images for: Involvement of the Microglial Aryl Hydrocarbon Receptor in Neuroinflammation and Vasogenic Edema after Ischemic Stroke
Source: Cells. 2021 Mar 24;10(4):718. doi: 10.3390/cells10040718 (PMC8063823; doi:10.3390/cells10040718)

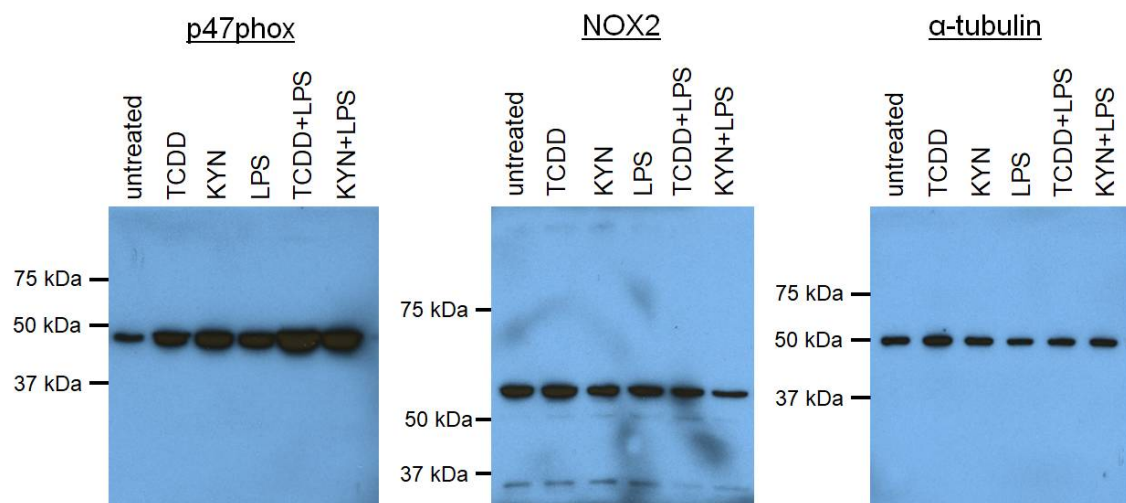

Figure S1. Whole membranes for the immunoblots presented in Figure 5B

Supplement: Supplementary file 1 [file cells-10-00718-s001.pdf]
